# Proteomic Analysis of Whole Human Saliva Detects Enhanced Expression of Interleukin-1 Receptor Antagonist, Thioredoxin and Lipocalin-1 in Cigarette Smokers Compared to Non-Smokers

**DOI:** 10.3390/ijms11114488

**Published:** 2010-11-09

**Authors:** Kala Jessie, Wei Wei Pang, Zubaidah Haji, Abdul Rahim, Onn Haji Hashim

**Affiliations:** 1 Department of Oral Biology, Faculty of Dentistry, University of Malaya, 50603 Kuala Lumpur, Malaysia; E-Mails: jessiekala7@yahoo.com (K.J.); zubaidar@um.edu.my (Z.H.A.R.); 2 University of Malaya Centre for Proteomics Research, Faculty of Medicine, University of Malaya, 50603 Kuala Lumpur, Malaysia; E-Mail: wwpang@um.edu.my; 3 Department of Molecular Medicine, Faculty of Medicine, University of Malaya, 50603 Kuala Lumpur, Malaysia

**Keywords:** saliva, proteome, smoker, biomarker

## Abstract

A gel-based proteomics approach was used to screen for proteins of differential abundance between the saliva of smokers and those who had never smoked. Subjecting precipitated proteins from whole human saliva of healthy non-smokers to two-dimensional electrophoresis (2-DE) generated typical profiles comprising more than 50 proteins. While 35 of the proteins were previously established by other researchers, an additional 22 proteins were detected in the 2-DE saliva protein profiles generated in the present study. When the 2-DE profiles were compared to those obtained from subjects considered to be heavy cigarette smokers, three saliva proteins, including interleukin-1 receptor antagonist, thioredoxin and lipocalin-1, showed significant enhanced expression. The distribution patterns of lipocalin-1 isoforms were also different between cigarette smokers and non-smokers. The three saliva proteins have good potential to be used as biomarkers for the adverse effects of smoking and the risk for inflammatory and chronic diseases that are associated with it.

## 1. Introduction

Cigarette smoking is the most preventable cause of addiction, sickness and mortality in the world. Death attributed to cigarette smoking is estimated to rise from 5.4 million in 2005 to 6.4 million by 2015 [1]. Chronic cigarette smoking is the single most important risk factor for lung and oral cancers, cardiovascular diseases, chronic obstructive pulmonary disease (COPD) and other tobacco related oral diseases, including periodontitis [2–7]. Cigarette smoke contains more than 60 carcinogens and around 4,000 chemicals, including bacteria-derived endotoxins, which are toxic to cells [8–10]. The risk of developing tobacco smoking-related diseases increases with the total exposure time to the cigarette smoke, which generally includes the number of cigarettes a person smokes each day and the number of years a person has been smoking [11].

The oral cavity is the first organ in the human body to be exposed to the cigarette smoke. The tobacco smoke alters normal homeostasis of the oral cavity, including the saliva’s antioxidant and other protective systems. This may lead to oral inflammatory diseases and oral cancers [12–15]. Early tumorigenic activities have been detected in normal oral mucosa of heavy smokers who have no overt precancerous or cancerous lesions [16]. The mucosal changes in smokers may also arise from the drying effects of the mucosa, high intraoral temperatures, intraoral pH changes, local alteration of membrane barriers and immune responses, or altered resistance to bacteria, fungal and viral infections. Smoking-related cell damage may leave molecular footprints in the saliva, offering the potential for non-invasive early diagnosis of tobacco-related oral diseases.

Human saliva contains a large number of proteins and peptides that are easily accessible and may serve as a potential source of biomarkers to monitor changes that occur under pathological conditions. The value of saliva as a biological fluid for the detection of diagnostic and prognostic biomarkers has become increasingly well established [17–24]. Collection of human saliva is a simple, non-invasive and cost-effective approach for screening large populations. It is easy to handle and may be repeated without inflicting much discomfort to the subjects [17,18].

Proteomic analysis is an important investigative tool used to systematically explore cellular proteins that are responsive to adverse environmental challenges. Several proteomic approaches, including those involving separation of proteins by two-dimensional electrophoresis (2-DE), have been applied in the investigation of biomarker candidates in the human saliva [25–29]. Recently, saliva has been shown to harbor potential informative biomarkers for oral cancer [30–32], head and neck cancer [33,34], and breast cancer [35]. While effects of the cigarette smoke on proteins expressed in the bronchoalveolar lavage [36–38], nasal lavage fluid [39], urine [40], lung tissue [41], bronchial airway epithelium and pooled exhaled breath condensate samples [42] have been analyzed, little information is available regarding the effects of smoking on the whole saliva proteome.

To the best of our knowledge, there had been no reported studies that specifically compared the expression of proteins in the saliva of smokers and non-smokers. In this study, 2-DE-based proteomics was used to screen for saliva proteins of differential abundance between smokers and subjects who had never smoked. The aberrantly expressed proteins, when correlated to those similarly altered in the saliva of patients with tobacco-related diseases including oral cancer, may potentially be used as biomarkers to indicate risks for the various diseases.

## 2. Materials and Methods

### 2.1. Collection of Whole Saliva

Unstimulated whole saliva samples were collected from 24 healthy Malay male volunteers aged between 35 and 55 years (12 smokers and 12 non-smokers), with no history of diabetes, autoimmune diseases or exposure to radiation and chemotherapy. Characteristics of the participants who were considered heavy smokers in this study are shown in Table 1. Saliva samples were collected with the volunteers’ consent and approval granted by the Ethical committee (Institutional Review Board) of the Faculty of Dentistry, University of Malaya. Each subject answered a questionnaire concerning personal data, smoking and alcohol drinking habits, health or dental problems, oral hygiene habits, previous dental examinations, use of prescriptions, quantity and length of cigarette smoking. Unstimulated whole saliva was collected in the morning between 9 and 11 am to minimize the circadian effect, and subjects refrained from eating, drinking, smoking or performing any oral hygiene for at least 2 h prior to the collection. The difference between the mean flow rates of non-smokers (0.39 ± 0.04 mL/min) and smokers (0.42 ± 0.04 mL/min) was not statistically significant. Protease inhibitor cocktail was added to the saliva immediately after collection as previously described [43]. To remove debris and cells, the saliva was centrifuged at 14,000 g for 20 min at 4 °C and the proteins were precipitated in 10% TCA/acetone/20 mM DTT. Saliva proteins were quantified using the Bradford protein assay kit (Bio-Rad, Hercules, USA) according to the manufacturer’s instructions.

### 2.2. Two-Dimensional Electrophoresis

Two-dimensional electrophoresis (2-DE) was performed as previously described [43]. Saliva proteins (130 μg) were dissolved in rehydration buffer containing 7 M urea, 2 M thiourea, 4% CHAPS, 0.5% IPG buffer, 65 mM DTT and 0.002% bromophenol blue and applied onto 13 cm rehydrated precast immobilized drystrips pH 4–7 (GE Healthcare BioSciences, Uppsala, Sweden). Isoelectric focusing (IEF) for the first dimension and SDS-PAGE for the second dimension were performed as described previously [43]. All samples were analyzed in triplicate.

### 2.3. Silver Staining

The 2-DE gels were developed by silver staining as described by Heukeshoven and Dernick [44]. For mass spectrometry analyses, gels were stained with compatible silver staining with slight modifications according to Yan *et al*. [45].

### 2.4. Image Analysis

The LabScan image scanner was used to capture and store images of 2-DE gels. The GE ImageMaster™ 2D Platinum Software version 7 was used to evaluate the protein profiles and perform protein analyses. To detect proteins that were differentially secreted in the saliva, the percentage volume contribution (% vol) of a protein spot, which refers to the spot volume of a protein expressed as a percentage of the total spot volume of all detected saliva proteins, was calculated. Data expressed this way are independent of variations attributed to protein loading and staining. The 2-DE profiles and relative spot intensities obtained were reproducible when performed in triplicate.

### 2.5. In Gel Trypsin Digestion and Mass Spectrometry

Highly resolved protein spots were initially identified by visual comparison with previously published protein maps obtained from the human whole saliva [22–26]. The protein spots (1–2 mm diameter) were excised from silver-stained gels with pipette tips and kept hydrated in clean microfuge tubes containing Milli-Q water, prior to the in-gel digestion. Trypsin digestion and precise identification by mass spectrometry, using the MALDI-TOF/TOF instrument (Applied Biosystem 4800 Proteomic Analyzer), were performed as previously described [46].

### 2.6. Database Searches

Spectra were processed and analyzed by the Global Protein Server Workstation (Applied Biosystems), which uses the internal MASCOT (Matrix Science, London, UK) software for search of the peptide mass fingerprints and MS/MS data. Searches were performed against the Swiss-Prot database (Last update: October 23, 2008, containing 261513 sequences). Database search parameters were set as follows: The enzyme trypsin was used; up to one missed cleavage was allowed; variable modification included were carbamidomethylation of cysteine and oxidation of methionine; the mass tolerance for MS precursor ion and MS/MS fragment ion were 100 ppm and 0.2 Da, respectively; and only monoisotopic masses were included in the search.

### 2.7. Statistical Analysis

All values are presented as mean ± S.E.M (standard error of the mean). The Student’s t-test was used to analyze the significance of difference between non-smokers and smokers. The false discovery rate control was performed using the method of Benjamini and Hochberg [47].

## 3. Results

**Figure 1.**
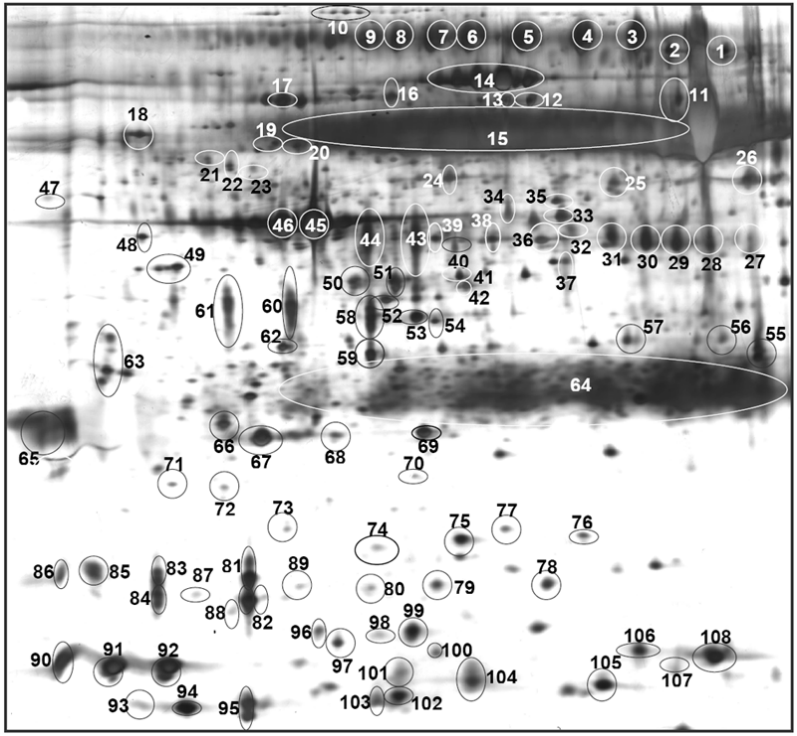
Typical 2-DE profile of precipitated saliva proteins obtained from non-smokers. A total of 108 protein spots (circled and numbered) were identified by mass spectrometry and database search (please refer to Table 2). Acid side of 2-DE gel is to the left and relative molecular mass declines from the top.

Figure 1 shows a typical 2-DE profile of saliva proteins separated between pH 4 and 7 in healthy non-smokers. This range of pH was chosen as our earlier 2-DE results performed at a pH range of 3 to 10 showed that most of the saliva proteins were located in the acidic region between pH 4 to 7. More than 120 spots were detected in the whole saliva samples using the 2-DE that was performed under the conditions of our study. Identities of 108 spots belonging to 57 different proteins were established by MS and database search (Table 2). Some of these proteins, including polymeric immunoglobulin receptor (spots 3–9), carbonic anhydrase VI (spots 27–32), prolactin inducible proteins (spots 81–86), zinc-alpha-2-glycoprotein (spots 43 and 44), short palate, lung and nasal epithelium carcinoma-associated protein 1 (spots 58–61), cystatin S (spots 90 and 91) and lipocalin-1 (spots 87–89) were resolved in several isoforms and thus separated into distinct spots in the 2-DE gels.

Among the total of 57 saliva proteins, 35 had been previously identified using 2-DE [32–39], whereas 16, including plastin-2, actin-related protein-3, C3 complement precursor, macrophage capping protein, F actin capping protein, annexin A3, protein kinase C inhibitor protein-1, rho-GDP-dissociation inhibitor 1, rho-GDP-dissociation inhibitor 2, actin-related protein 2/3 complex subunit 5, alpha-1-acid glycoprotein 1, chloride intracellular channel protein 1, protein disulfide-isomerase, leukotriene A-4 hydrolase, IgGFc-binding protein and long palate, lung and nasal epithelium carcinoma-associated protein 1, were previously detected using liquid-based proteomics [21,23]. The other six saliva proteins, eosinophil lysophospholipase, beta-microseminoprotein, coactosin-like protein, nucleoside diphosphate kinase A, calreticulin and synaptic vesicle membrane protein VAT-1, are reported for the first time by this study.

**Figure 2.**
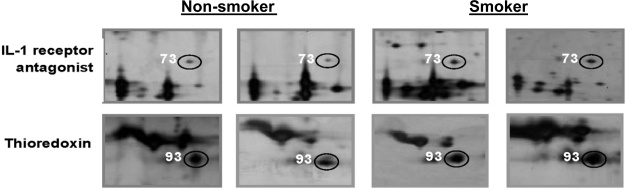
Cropped images of saliva proteins in the 2-DE gels of non-smokers and smokers. Representative gels of two differentially expressed saliva proteins are shown. Spot numbers are those referred to in Table 2.

When 2-DE was performed on whole saliva samples of heavy smokers, the profiles obtained were similar to those from non-smokers. All 57 different proteins that were expressed in the saliva of the non-smokers were also detected in the saliva of the heavy smokers although the rates of presence of 16 proteins in the 2-DE profiles of the cigarette smokers were different from those of the non-smokers. When the 2-DE protein profiles obtained from the non- and heavy smokers were subjected to densitometry analysis, initially a significantly enhanced expression of seven proteins including polymeric immunoglobulin receptor, complement C3, α1-antitrypsin, calgranulin B, interleukin-1 receptor antagonist, thioredoxin and lipocalin-1, was detected between the two subject groups. However, only three of the proteins, *i.e.*, interleukin-1 receptor antagonist (+3 fold), thioredoxin (+2.5 fold) and lipocalin-1 (+4.4 fold) were found to be truly significant when the *p*-values were corrected for false significant results using the method of Benjamini and Hochberg [47] (Table 3). Figure 2 demonstrates examples of 2-DE spot clusters of proteins whose levels were altered in the saliva obtained from the heavy smokers as compared to those of the non-smokers.

**Figure 3.**
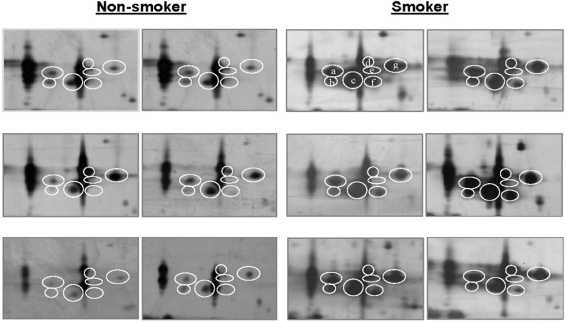
Cropped images of lipocalin-1 isoform spots in the 2-DE gels of non-smokers and smokers. Six representative gels are shown. The isoform spots a to g are marked in the gels (only represented in one of the images so as not to affect image display). Detailed densitometry analysis of the isoform spots is demonstrated in Table 4.

When the different isoforms of polymeric immunoglobulin receptor (spots 3–9), carbonic anhydrase VI (spots 27–32), prolactin inducible proteins (spots 81–86), zinc-alpha-2-glycoprotein (spots 43 and 44), short palate, lung and nasal epithelium carcinoma-associated protein 1 (spots 58–61) and cystatin S (spots 90 and 91) were similarly analyzed by densitometry, their volume distribution patterns were found to be consistent between the saliva of non-smokers and smokers. In contrast, the 2-DE volume distribution pattern for isoforms of lipocalin-1 in the saliva of non-smokers was different from that detected in the saliva of the heavy smokers (Figure 3). Among the seven isoforms analyzed, the isoform f was almost exclusive to the saliva of the smokers (Table 4).

## 4. Discussion

Human whole saliva contains fluid from the salivary glands, gingival crevicular fluid, bronchiol and nasal secretions, desquamated epithelial cells, oral tissues, and very often, the components of blood, bacteria and viruses [48–50]. Therefore, whole saliva—in contrast to serum—is a hostile environment with proteins subjected to the effects of many host- and bacteria-derived enzymes. Some saliva proteins are synthesized in the salivary glands and subsequently subjected to intracellular processing including glycosylation, phosphorylation and proteolysis. Once the secretions enter the non-sterile oral environment, additional and continuous protein modifications by host- and bacteria-derived enzymes occur. This results in the possible generation of many modified proteins in whole saliva [51].

The 2-DE profiles of proteins in whole saliva from healthy non-smokers that were generated in the present study showed strong resemblance to those that were previously reported [22–26]. Almost 90% of the protein spots that were highly resolved were eventually identified. The remaining spots were unidentifiable as the proteins generated low intensity spectra probably due to their low amounts, resistance to proteolytic cleavage, low recovery of digested peptides, and/or low efficiency in peptide ionization. Nevertheless, it is also possible that some of the unidentified proteins were of bacterial origin since the mouth is likely to harbor a lot of microorganisms.

In addition to the 35 human saliva proteins that have previously been established by other research groups using 2-DE [22–26], the present study detected the presence of 22 additional proteins. This is an important contribution to the human saliva proteome as a whole. Among the newly identified proteins (see Table 2), nucleotide diphosphate kinase A, annexin A3, Rho-GDP-dissociation inhibitor 1, beta-microseminoprotein, chloride intracellular channel protein 1, protein disulfide-isomerase, calreticulin, peroxiredoxin-2, alpha-1-acid glycoprotein 1 and IgG Fc-binding protein are considered clinically interesting as they have been previously associated with cancer and other diseases [52–61].

The establishment of highly resolved 2-DE protein profiles enabled investigations on protein changes associated with cigarette smoking. Densitometry analyses on the 2-DE protein profiles obtained from the non- and heavy smokers showed differential abundance of interleukin-1 receptor antagonist, thioredoxin and lipocalin-1 between the saliva samples of the two subject groups (Table 3). The three proteins have good potential to be used as non-specific complementary biomarkers for the adverse effects of smoking although this requires further evaluation and correlative studies. Some of the proteins may be used as risk indicators for inflammatory and chronic diseases that are associated with smoking as they have been shown to be of increased levels in the saliva of the patients. In the case of lipocalin-1, the isoform distribution pattern detected was also found to differ between smokers and non-smokers. This suggests that the carbohydrate moieties of lipocalin-1 of the heavy cigarette smokers were different from those of the non-smokers and that they may be differently glycosylated or modified. However, this remains to be further established.

Despite being distinctly categorized according to their primary biological roles [62], the three saliva proteins that were altered in abundance reflect the body’s overall response to the damaging effects of heavy smoking. The high levels of IL-1 receptor antagonist in the saliva of the heavy smokers detected in this study reflect an anti-inflammatory response in the oral cavities of the smokers. Increased generation of the proteins in smokers may be induced by the proinflammatory cytokines that were promoted by oxidative stress [63–65]. An imbalance between IL-1 receptor antagonist and IL-1 has been hypothesized to play a role in the pathogenesis of various inflammatory diseases [65].

Lipocalin-1 and thioredoxin are proteins most likely involved in the response to stress in relation to tissue damage. The high levels of lipocalin-1 and thioredoxin in the cigarette smokers’ saliva may reflect their function as an oxidative stress-induced scavenger against toxic and pro-inflammatory lipids [66–68]. Lipocalin-1 had been suggested to be a cysteine proteinase inhibitor [67] and may have a role in the control of inflammatory processes in oral tissues. Thioredoxin, on the other hand, was shown to modulate remodeling factors in response to the cigarette smoke [68]. Increased secretion of thioredoxin had been previously demonstrated in the saliva of patients with oral cancer [32].

## 5. Conclusion

Comparative proteomics analysis of human saliva samples from subjects who were considered heavy cigarette smokers and those who did not smoke detected altered abundance of interleukin-1 receptor antagonist, thioredoxin and lipocalin-1, as well as a change in the isoform distribution patterns of lipocalin-1. These proteins may be used as early biomarkers to indicate risks of tobacco-related diseases.

## Figures and Tables

**Table 1.** Demographics and smoking history of smoker subjects.

**Subject(a)**	**Age**	**Cigarettes/Day**	**Smoking Duration(b)**	**Stick-year(c)**
1	35	20	15	300
2	36	14	23	322
3	38	20	15	300
4	51	14	20	280
5	36	14	15	210
6	54	20	30	600
7	38	20	17	340
8	54	30	30	900
9	39	20	20	400
10	39	20	25	500
11	48	24	17	408
12	40	14	15	210

(a) All subjects were male and of Malay ethnicity;

(b) Duration in years since first started smoking;

(c) Stick-year of exposure is in accordance to the Brickman index, which is the number of cigarettes smoked per day multiplied by the smoking duration.

**Table 2.** Identification of saliva proteins by mass spectrometry.

**Accession No. (a)**	**Protein**	**Spot No.(b)**	**MASCOT Score**	**No. of Peptides Hit**	**Sequence Coverage**	**Ref.**
P02787	Serotransferrin	1	89	10	9	[22,23,25]
		2	78	16	8	
P01833	Polymeric immunoglobulin receptor	3	282	9	8	[22–26]
		4	111	11	33	
		5	99	17	27	
		6	240	7	22	
		7	246	20	55	
		8	169	12	32	
		9	120	18	28	
Q9Y6R7	IgGFc-binding protein	10	645	10	2	Npd
Q8TDL5	*Long palate, lung and nasal epithelium carcinoma-associated protein 1	11	187	4	11	Npd
P09960	*Leukotriene A-4 hydrolase	12	376	6	14	Npd
P02768	Serum albumin	13	98	3	5	[22–26]
		14	122	5	7	
P04745	Human salivay α-amylase	15	101	15	27	[22–26]
P08107	Heat shock 70 kDa protein 1	16	572	8	14	[23]
P13796	Plastin-2	17	555	32	16	Npd
P07237	*Protein disulfide-isomerase	18	636	13	27	Npd
P02774	Vitamin D-binding protein precursor	19	741	11	32	[27]
		20	575	10	27	
P01009	Alpha-1-antitrypsin	21	211	8	10	[25]
		22	112	6	12	
		23	99	5	18	
P61158	Actin-related protein 3	24	327	13	17	Npd
P50395	*Rab GDP dissociation inhibitor beta	25	363	15	19	[28]
P06733	Alpha-enolase	26	735	11	33	[22,23,26]
P23280	Carbonic anhydrase VI	27	290	15	19	[22,23]
		28	90	4	4	
		29	303	11	40	
		30	488	32	48	
		31	79	4	8	
		32				
P30740	*Leukocyte elastase inhibitor	33	374	12	19	[29]
		34	315	10	16	
Q99536	**Synaptic vesicle membrane protein VAT-1	35	76	2	4	Npd
P40121	Macrophage-capping protein	36	579	16	20	Npd
P02675	Fibrinogen beta chain	37	676	12	26	[23,25]
		38	554	16	36	
		39	467	15	20	
P00738	Haptoglobin	40	518	10	21	[28]
P37837	*Transaldolase	41	60	8	15	[28]
		42	77	6	13	
P25311	Zinc-alpha-2-glycoprotein	43	246	16	28	[22–26]
		44	285	11	20	
P60709	Actin, cytoplasmic 1	45	230	3	15	[23,25]
		46	188	4	15	
P27797	**Calreticulin	47	651	10	27	Npd
P01024	Complement C3	48	330	15	6	Npd
		49	332	14	5	
P63261	Actin, cytoplasmic 2	50	285	11	20	[23]
P60709	Actin, cytoplasmic 1	51	243	5	14	[23,25]
P52907	F-actin-capping protein subunit alpha-1	52	274	6	16	Npd
P25311	Zinc-alpha-2-glycoprotein	53	82	3	10	[22–26]
P12429	Annexin A3	54	331	8	14	Npd
P00738	Haptoglobin	55	627	12	24	[28]
P01876	Ig alpha-1 chain C region	56	274	5	17	[22–24,26]
P30740	*Leukocyte elastase inhibitor	57	417	20	30	[29]
Q96DR5	*Short palate, lung and nasal epithelium carcinoma-associated protein 2	58	141	4	15	[22]
		59	119	5	18	
		60	293	20	68	
		61				
O00299	*Chloride intracellular channel protein 1	62	561	9	52	Npd
P63104	Protein kinase C inhibitor protein-1(14-3-3 protein zeta/delta)	63	101	4	12	Npd
P01834	Ig kappa chain C region	64	101	4	33	[23–26]
P01591	Immunoglobulin J chain	65	241	11	32	[22,24,25]
P52565	Rho GDP-dissociation inhibitor 1	66	356	13	30	Npd
P52566	*Rho GDP-dissociation inhibitor 2	67	173	9	31	Npd
P09211	Glutathione S Transferase	68	201	6	22	[22–24,26]
		69	493	19	61	
P32119	Peroxiredoxin-2	70	264	6	27	[28]
P02763	Alpha-1-acid glycoprotein 1	71	317	6	27	Npd
		72	137	4	17	
P18510	Interleukin-1 receptor antagonist protein	73	148	6	15	[22,24]
P00738	Haptoglobin	74	358	6	22	[28]
		75	491	7	20	
		76	439	8	22	
P15531	**Nucleoside diphosphate kinase A	77	132	6	25	Npd
P52566	Rho GDP-dissociation inhibitor 2	78	185	4	32	Npd
O15511	Actin-related protein 2/3 complex subunit 5	79	69	2	7	Npd
P12273	Prolactin-inducible protein	80	385	6	45	[22–25]
		81	116	32	6	
		82	375	5	44	
		83	365	6	45	
		84	410	6	45	
		85	413	7	54	
		86	279	5	45	
P31025	*Lipocalin-1	87	169	5	18	[22,24]
		88	168	3	17	
		89	213	4	21	
P01036	*Cystatin S	90	535	15	62	[22–24,26]
		91	444	8	58	
P09228	*Cystatin SA	92	437	7	64	[22–26]
P10599	Thioredoxin	93	88	4	16	[21]
		94	111	3	15	
P12273	Prolactin-inducible protein	95	413	7	54	[22–25]
P02766	Transthyretin	96	80	3	33	[28,33]
Q14019	**Coactosin-like protein	97	379	8	60	Npd
P08118	**Beta-microseminoprotein	98	119	2	8	Npd
P02766	Transthyretin	99	269	7	40	[28,33]
Q01469	*Fatty acid-binding protein, epidermal	100	219	6	47	[22–24,29]
P01036	*Cystatin S	101	192	4	37	[22–24,26]
P06702	Calgranulin-B	102	296	6	51	[22,24,26]
P01040	Cystatin-A	103	42	1	18	[22,25,26]
P06702	Calgranulin-B	104	395	7	63	[22,24,26]
P28325	*Cystatin D	105	172	6	34	[22,24]
Q01469	*Fatty acid-binding protein, epidermal	106	347	8	52	[22–24,29]
Q05315	**Eosinophil lysophospholipase	107	108	3	9	Npd
P01037	*Cystatin SN	108	293	20	68	[22–26]

(a) Accession no. are in accordance to Swiss-Prot;

(b) Spot numbers are those referred to in Figures 1 and 2 and identified by MS/MS; Npd—proteins not previously detected in the saliva proteome using 2-DE;

* Proteins found only in saliva and not in plasma;

** Proteins detected for the first time in the saliva proteome of this study.

**Table 3.** Densitometry analysis of saliva proteins and their rates of presence in 2-DE profiles.

**Protein**	**Non-smokers**		**Smokers**		

	**% volume(a)(±S.E.M)**	**RP(b)/12**	**% volume(a) (±S.E.M)**	**RP(b)/12**	***p*(c)**
**1: Energy/Metabolism**					
Amylase	14.15 (±0.54)	12	12.72 (±0.85)	12	0.168
Carbonic anhydrase VI	1.48 (±0.17)	12	1.07 (±0.14)	12	0.077
Zinc-alpha-2-glycoprotein	1.02 (±0.09)	12	1.09 (±0.13)	12	0.673
Fatty acid-binding protein, epidermal	0.08 (±0.01)	12	0.12 (±0.02)	12	0.111
Transaldolase	0.04 (±0.01)	12	0.04 (±0.01)	12	0.543
Alpha-enolase	0.06 (±0.02)	10	0.10 (±0.02)	12	0.183
**2: Defence/Immune response**					
Polymeric immunoglobulin receptor	4.58 (±0.12)	12	3.57 (±0.40)	12	0.024
Immunoglobulin J chain	0.38 (±0.06)	12	0.41 (±0.06)	12	0.305
Interleukin-1 receptor antagonist protein	0.01 (±0.00)	7	0.04 (±0.01)	11	0.004
Prolactin-inducible protein	2.27 (±0.20)	12	2.35 (±0.23)	12	0.787
Short palate, lung and nasal epithelium carcinoma-associated protein 2	1.22 (±0.18)	11	1.42 (±0.18)	12	0.456
Alpha-1-acid glycoprotein 1	0.01 (±0.00)	2	0.02 (±0.01)	5	0.159
**3: Protein degradation inhibitor**					
α_1_-Antitrypsin	0.02 (±0.01)	6	0.05 (±0.01)	8	0.027
Cystatin A	0.08 (±0.02)	12	0.09 (±0.03)	12	0.620
Cystatin S	0.04 (±0.01)	12	0.05 (±0.01)	12	0.775
Cystatin SA	0.28 (±0.05)	12	0.27 (±0.07)	12	0.916
Cystatin SN	0.28 (±0.06)	12	0.30 (±0.04)	12	0.744
Cystatin D	0.12 (±0.02)	12	0.12 (±0.02)	11	0.769
Leukocyte elastase inhibitor	0.10 (±0.01)	12	0.10 (±0.01)	12	0.922
**4: Cell adhesion/communication**					
Calgranulin B	0.13 (±0.01)	12	0.21 (±0.04)	12	0.032
**5: Protein folding/repair**					
Heat shock 70 kDa protein 1	0.06 (±0.01)	12	0.05 (±0.01)	12	0.818
**6: Redox**					
Thioredoxin	0.03 (±0.00)	1	0.07 (±0.02)	8	0.001
Peroxiredoxin-2	0.01 (±0.01)	3	0.02 (±0.01)	7	0.617
**7: Signaling**					
Complement C3 precursor	0.00 (±0.00)	3	0.01 (±0.00)	9	0.012
Glutathione-S Transferase	0.10 (±0.01)	12	0.12 (±0.02)	12	0.444
Rho GDP-dissociation inhibitor 1	0.14 (±0.03)	3	0.15 (±0.03)	3	0.557
Rho GDP-dissociation inhibitor 2	0.03 (±0.01)	10	0.04 (±0.01)	12	0.327
Protein kinase C inhibitor protein-1	0.11 (±0.03)	7	0.17 (±0.05)	11	0.282
Annexin A3	0.07 (±0.02)	11	0.00 (±0.01)	6	0.088
**8: Structural/cytoskeletal**					
f-actin-capping protein subunit alpha-1	0.02 (±0.01)	4	0.03 (±0.01)	8	0.521
Macrophage-capping protein	0.03 (±0.01)	7	0.02 (±0.00)	9	0.261
l-plastin	0.11 (±0.01)	12	0.09 (±0.01)	12	0.376
**9: Transport**					
Lipocalin-1	0.15 (±0.05)	8	0.65 (±0.13)	12	0.001
Haptoglobin	0.06 (±0.01)	11	0.07 (±0.01)	12	0.694
Transthyretin	0.07 (±0.01)	12	0.10 (±0.02)	12	0.108
Serum albumin	1.63 (±0.14)	12	1.71 (±022)	12	0.758

(a) volume of a protein expressed as a percentage of the total spot volume of all proteins;

(b) rate of presence of the protein spots in the 12 2-DE profiles that were analyzed;

(c) *p*-values of less than 0.0068 (*p* < 0.0068) were considered statistically significant when the false discovery rate procedure of Benjamini and Hochberg [47] was performed to the data set.

**Table 4.** Densitometry analysis of lipocalin-1 isoforms and their rates of presence in 2-DE profiles.

**Isoform Spot(a)**	**Non-smokers**		**Smokers**		***p***	**Fold Change(d)**

	**% vol(b)**	**RP(c)**	**% vol(b)**	**RP(c)**		
a	0.056	8	0.195	12	0.005	+3.5
b	0.007	4	0.150	10	0.000	+21.4
c	0.085	11	0.305	12	0.012	+3.7
d	0.013	5	0.108	11	0.012	+8.3
e	0.006	4	0.069	9	0.038	+11.5
f	0.003	1	0.089	6	0.031	+29.7
g	0.110	12	0.316	12	0.018	+2.9

(a) isoforms of lipocalin-1 as depicted in Figure 3;

(b) volume of a protein expressed as a percentage of the total spot volume of all proteins;

(c) rate of presence of the protein spots in the 12 2-DE profiles that were analyzed;

(d) fold change is the ratio of %vol of smokers to non-smokers.
